# Systematic reviews and cancer research: a suggested stepwise approach

**DOI:** 10.1186/s12885-018-4163-6

**Published:** 2018-03-02

**Authors:** George A. Kelley, Kristi S. Kelley

**Affiliations:** School of Public Health, Department of Biostatistics, Robert C. Byrd Health Sciences Center, West Virginia University, PO Box 9190, Morgantown, WV 26506-9190 USA

**Keywords:** Cancer, Systematic review, Meta-analysis, Guidelines, Methods

## Abstract

Systematic reviews, with or without meta-analysis, play an important role today in synthesizing cancer research and are frequently used to guide decision-making. However, there is now an increase in the number of systematic reviews on the same topic, thereby necessitating a systematic review of previous systematic reviews. With a focus on cancer, the purpose of this article is to provide a practical, stepwise approach for systematically reviewing the literature and publishing the results. This starts with the registration of a protocol for a systematic review of previous systematic reviews and ends with the publication of an original or updated systematic review, with or without meta-analysis, in a peer-reviewed journal. Future directions as well as potential limitations of the approach are also discussed. It is hoped that the stepwise approach presented in this article will be helpful to both producers and consumers of cancer-related systematic reviews and will contribute to the ultimate goal of preventing and treating cancer.

**Electronic supplementary material:**

The online version of this article (10.1186/s12885-018-4163-6) contains supplementary material, which is available to authorized users.

## Background

**Fig. 1:**
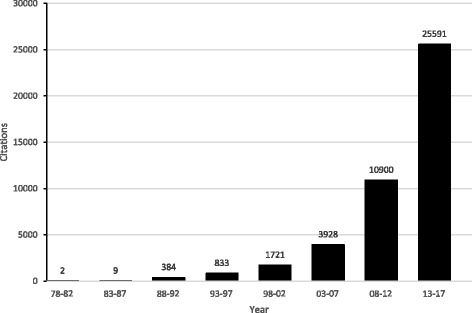
Results of PubMed search for systematic reviews, with or without meta-analysis, in the field of cancer up to November 7, 2017

**Fig. 2:**
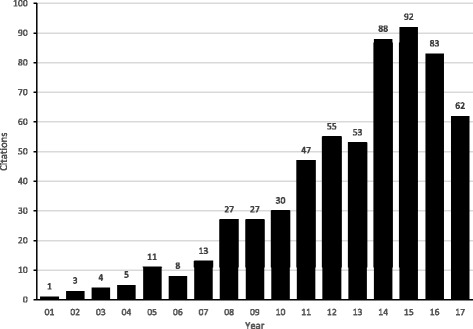
Results of PubMed search for systematic reviews, with or without meta-analysis, limited to the journal *BMC Cancer* up to November 8, 2017

Given the proliferation of original studies on the same topic, often with varying results, systematic reviews, with or without meta-analysis, play an important role today in evidence synthesis. As an example of the importance of this approach with respect to the sheer volume of information, it has been reported that in 1992, a primary care physician would need to read 17 research articles per day, 365 days per year, to stay current in her/his field [1], something that is obviously not realistic. Using a rigorous and systematic approach to reviewing the literature provides one with a more valid and reliable consolidation of information regarding the topic of interest. If a meta-analysis is included as part of a systematic review, such analyses can (1) increase statistical power for primary outcomes as well as subgroups, (2) address uncertainty when study results differ, (3) improve estimates of effect size, and (4) answer questions not established at the start of individual trials [2]. In the field of cancer, the number of systematic reviews, with or without meta-analysis, has increased dramatically over the past 30 years. For example, a recent PubMed search conducted by the authors on November 7, 2017 and limited to systematic reviews, with or without meta-analysis in the field of cancer yielded two citations for the five-year period 1978 through 1982 compared to 25,591 citations from 2013 through 2017 (Fig. 1). Specific to *BMC Cancer*, the number of indexed citations have increased from 24 for the five-year period 2001 through 2005 to 378 for the years 2013 through 2017 (Fig. 2). Given the dramatic increase in the number of systematic reviews in cancer research, we are now faced with multiple systematic reviews, with or without meta-analysis, on the same topic. As a result, there is now a need to conduct systematic reviews of previous systematic reviews (SRPSR), with or without meta-analysis, in order to provide decision-makers with the “state-of-the evidence” as well as researchers of original studies and systematic reviews with direction for both the conduct and reporting of future research, including information on whether an original or updated systematic review on the topic of interest is needed [3, 4]. This latter factor may be especially relevant given the recent criticism regarding the production of redundant and unnecessary systematic reviews on the same topic [5]. As an example of a SRPSR that was limited to studies that conducted meta-analyses, the authors recently published a study in *BMC Cancer* on exercise and cancer-related fatigue in adult cancer patients and survivors [6]. From the 16 systematic reviews with meta-analyses that included up to 3245 participants, it was concluded that a lack of certainty currently exists regarding the benefits of exercise on cancer-related fatigue in adult cancer patients and survivors [6]. It was suggested that while additional research is needed, exercise did not appear to increase cancer-related fatigue. Unfortunately, and to the best of the author’s knowledge, there is a lack of consolidated and practical guidance on the entire systematic review process, starting with the idea of conducting a SRPSR and possibly ending with an updated or new systematic review, with or without meta-analysis. The purpose of this paper is to try and help fill that gap.

### Suggested guidelines for the systematic review process

#### Overview

**Fig. 3:**
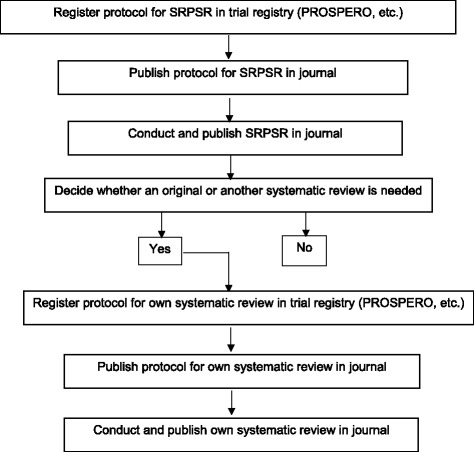
Suggested stepwise approach for the systematic review process

Given the proliferation of original studies as well as systematic reviews, with or without meta-analysis, on the same topic, it would appear plausible to suggest that one first conduct a SRPSR, with or without meta-analysis, prior to making any decision regarding the conduct of an original or updated systematic review on that topic. While previous guidelines for conducting a SRPSR, also known as ‘umbrella reviews’, ‘overviews of reviews’, ‘reviews of reviews’, ‘summary of systematic reviews’, ‘synthesis of reviews’, and ‘meta-reviews’, have been suggested [4, 7, 8], there appears to be a lack of detail as well as stepwise guidance in one document regarding (1) the process for conducting and publishing a SRPSR, (2) the decision-making process for whether an original or updated systematic review, with or without meta-analysis, is needed, and (3) the process for conducting and publishing one’s own systematic review, with or without meta-analysis. In this article, the authors draw upon their own experiences as well as previous research to address this gap, including a practical, stepwise approach for systematically reviewing the literature and publishing the results. Broadly, this process tentatively starts with the registration of a protocol for a SRPSR and possibly ends with the publication of an original systematic review, with or without meta-analysis, in a peer-reviewed journal. A description of the proposed stepwise approach is shown in Fig. 3 with a more detailed, but not exhaustive, description that follows. For the current article, the focus is on the systematic review process as applied to studies relevant to cancer in health care settings, although much of this information can be applied to other health conditions and settings. References and additional files that provide more detailed information on current methods for reporting and conducting systematic reviews, with or without meta-analysis, are included as well as a revised checklist specific to SRPSR. It is the hope that this stepwise document will serve as a guide to both producers (authors and journal editors), consumers (researchers, health care practitioners, guideline developers) and funding agencies with respect to the process for producing high-quality systematic reviews that have a meaningful impact on the field of cancer.

### Step 1. Register protocol for systematic review of previous systematic reviews, with or without meta-analysis, in trial registry

Similar to clinical trials as well as original systematic reviews, with or without meta-analysis, registering a protocol prospectively for a SRPSR in a systematic review trial registry such as the International Prospective Register of Systematic Reviews (PROSPERO, https://www.crd.york.ac.uk/prospero/) is an important first step when conducting a SRPSR, with or without meta-analysis. The assumptions here are that no previous SRPSR exist on the topic of interest but previous and original systematic reviews do exist. This determination should be made based on a preliminary search of PROSPERO as well as other databases, for example PubMed, since all SRPSR as well as original systematic reviews, are not registered in PROSPERO. If no previous systematic reviews exist, one could then move on to developing and registering the protocol for one’s own systematic review, with or without meta-analysis, assuming sufficient rationale is provided for such. A search of the PROSPERO trial registry on November 11, 2017 using the keyword “cancer” yielded 3615 registered protocols. While PROSPERO was originally intended for an original systematic review, with or without meta-analysis, they also allow for registration of SRPSR [6]. Generally, registration for a SRPSR should take approximately 30 min to complete and includes specific items to address, some of which may not be applicable to one’s own SRPSR [9]. Ideally, trial registration should occur prior to screening studies that meet one’s inclusion criteria for a SRPSR [9]. When reporting the proposed electronic database search strategies planned, one should make sure to include the terms, or some form (s) of the terms, ‘systematic review’ and ‘meta-analysis’. This will help reduce the number of false positives when conducting searches for previous systematic reviews. Similar to original systematic reviews, the major reasons for registration, described in detail elsewhere [9], include avoiding bias when conducting a review as well as avoiding unintended duplication of effort [5]. The registration of a SRPSR can benefit a number of entities. These include (1) researchers by making others aware of their work, (2) commissioning and funding organizations by avoiding unplanned duplication of effort, (3) guideline developers in the planning and development of guidelines, (4) journal editors as a protective measure against reporting biases as well as a means to enhance the peer review process, and (5) methodologists by providing information about how researchers design, conduct and report their SRPSR [9].

### Step 2. Publish protocol for SRPSR, with or without meta-analysis, in journal

The next step would be to submit one’s own SRPSR protocol for publication consideration in a peer-reviewed journal. For example, both *BMJ Open* and *Systematic Reviews* currently allow for the submission of protocols for publication consideration, and it is suggested that others, including *BMC Cancer*, allow for the same. The submission of a protocol for publication consideration can increase awareness of the planned study above and beyond PROSPERO registration as well improve the protocol based on feedback from reviewers and editors. In addition, this allows one to provide greater detail than that afforded in a trial registry such as PROSPERO, although at least a tertiary description of all planned activities should be reported in a trial registry. If the review is modified based on feedback from reviewers, the authors should go back and amend their protocol in PROSPERO or whatever registry in which the review is registered. When submitting one’s SRPSR protocol for publication consideration in a journal, the trial registry number should be included. In addition, a protocol checklist for one’s SRPSR should be included. Unfortunately, while previous guidelines for the conduct and reporting of a SRPSR have been suggested [4, 7], the authors are not aware of any protocol-based checklists for a SRPSR similar to the Preferred Reporting Items for Systematic Reviews and Meta-Analyses (PRISMA) protocol checklist (PRISMA-P) [10, 11]. Given the former, a suggested PRISMA-based protocol checklist for a SRPSR, developed by the authors and termed PRISMA-SRPSR-P, can be found in Additional file 1. The inclusion of a protocol checklist can benefit reviewers, editors, and authors in ensuring that all relevant items of a SRPSR are adequately addressed. For authors, the checklist inextricably leads back to both the appropriate conduct and reporting of a SRPSR.

Broadly, the authors believe that the three primary aims of a SRPSR, with or without meta-analysis, are to (1) provide a summary of the overall results from each included systematic review, with or without meta-analysis, as well as possibly conducting additional statistical analyses, (2) determine the quality and strength of evidence of the prior reviews, and (3) determine whether or not an original or updated systematic review, with or without meta-analysis is needed. We describe the first two aims below with a separate detailed description for aim three in step 4.

For aim 1, summarizing the overall results from a previous systematic review of systematic reviews, with or without meta-analysis, may be described at two levels, (1) a description of the characteristics of the SRPSR itself, and (2) a description of the characteristics of each included systematic review. For the former, items to report include such things as the number of previous systematic reviews that meet one’s inclusion, as well as exclusion, criteria. For each included systematic review, the presence or absence of a trial registration number should be recorded and reported as well as the methods used to assess for risk of bias in the original studies that were included. In addition, a study characteristics table along with a more detailed description in the text should be planned. Information to provide should include, but not necessarily be limited to, the following: (1) the reference for each previous systematic review, including the year in which the review was published, (2) the country in which the review was conducted, (3) the number of studies and participants included, including the sex of the participants, (4) the types of participants included, for example, breast cancer, colorectal cancer, etc., (5) the types of interventions, if any, that were included, and (6) the method (s) of assessment for the primary outcome (s) of interest in the original systematic review. If a meta-analysis was included, data to report should include, but not necessarily be limited to (1) the statistical methods used in the original systematic review to calculate effect sizes (original metric, standardized mean difference, odds ratios, relative risks, etc.), (2) statistical methods used to pool results (random-effects, inverse heterogeneity, confidence intervals, prediction intervals, etc.), (3) methods used for the assessment of heterogeneity and inconsistency (Q statistic, I-squared, meta-regression, influence analysis), (4) methods used to examine for small-study effects (quantitative tests, funnel plots, etc.) and (5) cumulative meta-analysis. While previous guidelines for conducting a SRPSR do not recommend such [4], one might also be interested in describing, a priori, any additional planned analyses that might enhance the robustness and applicability of findings not reported in the original meta-analyses. These may include such things as prediction intervals [12, 13], influence analysis, cumulative meta-analysis [14], percentile improvement [15, 16] and number needed to treat (NNT) [7, 17–20]. In addition, the authors might be interested in pooling results separately for each individual meta-analysis using pooling models that they may consider to be more robust than those used in the original meta-analyses. Furthermore, it is suggested that producers of SRPSR recalculate the results of all meta-analyses included to ensure that no errors were made in the original meta-analyses. Finally, one might also be interested in pooling the results from each study nested within each meta-analysis into one ‘mega’ meta-analysis [21]. However, if this is done, care should be taken to ensure that the same original study is not included more than once since the original meta-analyses included most likely were comprised of some of the same studies.

A second suggested aim is to assess the quality and/or risk of bias of each systematic review, with or without meta-analysis, as well as the strength of evidence of each review. One commonly used instrument for assessing the quality of systematic reviews, with or without meta-analysis, is A MeaSurement Tool to Assess systematic Reviews (AMSTAR), an 11-item instrument in which responses are coded as either ‘yes’, ‘no’, ‘can’t answer’ or ‘not applicable’ [22–24]. To asses for risk of bias, a recent instrument known as the Risk of Bias in Systematic Reviews (ROBIS), has been developed [25]. This instrument is completed in three phases: (1) assessing relevance (optional), (2) identifying concerns with the review process, and (3) judging the risk of bias [25].

In addition to assessing the quality and/or risk of bias of each included systematic review, one may also want to examine the strength of evidence from each systematic review. One commonly used instrument is The Grading of Recommendations, Assessment, Development and Evaluations (GRADE) instrument [26]. This tool evaluates the certainty of evidence for each pre-specified outcome [26]. Levels of evidence are rated as high, moderate, low or very low [27]. A study’s initial ranking is established by the study design but may be increased or decreased depending on other factors that include (1) risk of bias, (2) inconsistency, (3) indirectness, (4) imprecision, (5) publication bias, (6) size of the effect, (7) dose-response, and (8) residual confounding [27]. For SRPSR that include or focus on network meta-analyses [28], an alternative instrument based on the GRADE methodology has also been developed [29]. Based on the currently available evidence, it is suggested that the GRADE instrument be used to make decisions about the results of previous systematic reviews with meta-analyses that lead to different results and/or conclusions regarding such things as the effects of an intervention on the outcome of interest. Details regarding GRADE are described elsewhere [27].

### Step 3. Conduct and publish SRPSR, with or without meta-analysis, in a journal

After registering the protocol for a SRPSR in a trial registry such as PROSPERO and publishing the protocol in a peer-reviewed journal, the next step would be to actually conduct the SRPSR, with or without meta-analysis, and submit it for publication consideration in a peer-reviewed journal. Similar to the protocol for a SRPSR, no PRISMA guidelines or checklists currently exist for reporting the results of a SRPSR. Therefore, a suggested PRISMA-based checklist for a SRPSR, developed by the authors and termed PRISMA-SRPSR, is shown in Additional file 2. Assuming that the protocol for a SRPSR was published in a peer-reviewed journal, another potential issue has to do with self-plagiarism given that the rationale and methods will have already been published. However, this should only be an issue if authors fail to disclose to editors the existence of previous, related publications and do not explain the reasons for any overlap and/or editors fail to read the plagiarism software’s similarity report properly. Because plagiarism software calculates a percentage of similarities, this also needs to be checked manually to determine the reason for any potentially high scores. To minimize the perception of self-plagiarism, a cover letter to the editor should include a statement and citation for the previously published protocol along with a description of the extent of the overlap between the protocol and the submitted SRPSR. In addition, the protocol should be referenced in the Methods section of the submitted paper.

### Step 4. Decide whether another systematic review, with or without meta-analysis, is needed

The third aim of a SRPSR, with or without meta-analysis, is a decision about whether another systematic review, with or without meta-analysis, is needed. Given potential confusion and the lack of a true consensus on what constitutes an updated versus new systematic review, the term ‘another’ may be preferred. If no previous systematic review, with or without meta-analysis, has been identified, one may then conduct an original one (see steps 5-7). While there is currently no consensus on the one best approach for determining when another systematic review should be conducted, at least three different groups have provided guidelines for such, all with an organizational versus individual author (s) focus [30–32]. The Cochrane Collaboration currently recommends that another systematic review be based on needs and priority [30]. This decision is based on three primary factors: (1) strategic importance, (2) practical aspects with respect to organizing the review, and (3) impact of another review [30]. A decision is then made to either make the review a priority, postpone the review, or to no longer require such. In the United States, the Agency for Healthcare Research and Quality takes a needs-based approach to another review that focuses on stakeholder impact as well as the currency and need for the review [31]. Based on these general criteria, a decision is made to either create, archive, or continue surveillance [31]. More recently, the Panel for Updating Guidance for Systematic Reviews (PUGS) developed a consensus and checklist for when and how to conduct another systematic review [32]. This process includes (1) assessing the currency of the review, assuming one exists, (2) identifying relevant new methods, studies, or other information that may warrant another review, and (3) assessing the potential effect of another review [32]. From the authors’ perspective, the PUGS guidelines and checklist may be the most appropriate approach for researchers interested in conducting another systematic review, with or without meta-analysis.

### Step 5. Register protocol for own systematic review, with or without meta-analysis in trial registry

If a decision is made that another systematic review, with or without meta-analysis, is needed, the next suggested step would be to develop and submit one’s protocol to a systematic review registry such as PROSPERO. The reasons for this are similar to those described for registering the protocol for a SRPSR, with or without meta-analysis. In addition, one should reference and describe the previous SRPSR to help justify the need for another systematic review, with or without meta-analysis. Furthermore, authors should determine, a priori, whether they plan on including a meta-analysis in their systematic review. Usually, a meta-analysis should be planned for a systematic review with the protocol amended if one cannot be conducted.

### Step 6. Publish protocol for own systematic review, with or without meta-analysis, in journal

The next logical step would be to submit the completed protocol, including the trial registration number, to a peer-reviewed journal for publication consideration. Steps 6 and 7 are similar to previous steps 2 and 3 except now the focus is on retrieving individual studies to include in a systematic review rather than reviewing multiple systematic reviews. In addition to the protocol, it is suggested that authors include, and editors require, that the previously developed PRISMA protocol checklist be included (Additional file 3). A detailed description of the items in the checklist can be found elsewhere [10, 11]. The inclusion of a protocol will aid authors, reviewers and editors with respect to the work proposed. When writing the protocol, it is also important to provide a rationale for the methods one plans to use.

### Step 7. Conduct and publish own systematic review, with or without meta-analysis, in journal

The next step in the process is to conduct and publish one’s own systematic review, with or without meta-analysis. When submitting the manuscript for publication consideration, authors should also include, and journals should require, the inclusion of the appropriate PRISMA checklist, depending on the type of review conducted. This will also aid in the conduct of the review itself. Additional files 4, 5, and 6 include the PRISMA Checklists for an aggregate data meta-analysis, network meta-analysis, and individual participant data meta-analysis, respectively, with elaboration on these items described elsewhere [33–38]. Any previously published work (protocol for SRPSR, SRPSR study itself, protocol for own systematic review) should also be cited as well as all relevant protocol registration numbers. In addition, the issue of potential self-plagiarism should be handled similar to that described in step 3. If the protocol for one’s own systematic review has been published, both the Introduction and Methods sections should be similar to what was published in the original protocol. However, the Introduction and Methods section may warrant updating based on new information since the protocol was published. Any changes to the Methods section may require updating the registered protocol. What will be new are the Results, Discussion and Conclusions sections of the paper. The information reported in the Results section should be based on the planned analyses. If any type of analysis to explain heterogeneity is reported, for example meta-regression, it should be clearly pointed out in the Discussion section that because studies are not randomly assigned to covariates in meta-analysis, they are considered to be observational in nature. Consequently, the results do not support causal inferences. Rather, they should be considered as hypothesis-generating and thus, tested in original studies. In addition, because most meta-analytic studies use aggregate data and conduct a large number of statistical tests, it should be pointed out that there is the possibility that some statistically significant findings could have been nothing more than the play of chance. This is of course assuming that no adjustments for multiple testing were made. Finally, items to include in the Discussion section of one’s article could include (1) a description of the overall findings of the review, (2) implications for research, (3) implications for practice, (4) implications for policy, and (5) strengths and limitations of the review. The Conclusions section could briefly state the main findings of the review as well as any need for future research on the topic examined.

### Future directions

The information provided in this document provides suggested guidance starting with the potential conduct of a SRPSR and possibly ending with one’s own systematic review, with or without meta-analysis. In the future, it is expected that one will be faced with multiple SRPSR’s, thus creating another level of synthesis in cancer research as well as other areas. From a synthesis perspective, aggregate data meta-analyses with pairwise comparisons will probably continue to be the most common type of meta-analysis in the field of cancer as well as other fields. However, while it is anticipated that the number of individual participant data meta-analyses will continue to increase in the future, the authors do not expect that they will ever exceed the number of aggregate data meta-analyses because of the increased resources required as well as challenges in obtaining individual participant data from investigators. For example, the costs associated with an individual participant data meta-analysis of 11 studies on oral contraceptive use and the risk for ovarian cancer was reported to be $259,300, approximately five times the costs of an aggregate, i.e., summary data meta-analysis [39]. With respect to the availability of individual participant data, Nevitt et al., recently reported that only 25% of published IPD meta-analyses had access to all IPD [40]. Given the potential benefits with respect to treatment decisions, it is also expected that future meta-analyses will rely more heavily on network meta-analysis. This approach will allow for the integration of multiple treatments based on direct and indirect evidence for the outcome (s) of interest [41–44]. For those meta-analyses that include two or more dependent variables that are correlated, for example, sleep and fatigue in cancer patients [45], there is also expected to be an increase in the use of multivariate meta-analyses [42, 46]. Furthermore, it is expected that future meta-analytic research will include both network and multivariate meta-analysis so that both multiple treatments and outcomes can be examined in the same analysis [42, 47–51]. Finally, an area of increasing meta-analytic research has to do with genetic association studies. A PubMed search of meta-analytic genetic association citations limited to cancer up to November 8, 2017 demonstrated an increase from 12 for the five-year period 1992 to 1996 to 2684 for the 2013 to 2017 period. Issues and methods related to the specific conduct of meta-analysis for genetic association studies have been previously described in detail elsewhere [52–57].

### Limitations of suggested approach

One of the potential limitations of this stepwise approach is the time lag involved. For example, while obtaining a registration number for a protocol in PROSPERO occurs rather quickly, usually within a week based on the authors’ experience, the submission, review, and eventual publication of the protocol and article for both a SRPSR as well as an actual systematic review, with or without meta-analysis would most likely take several months each. As a result, and despite registering one’s protocol (s) in a trial registry such as PROSPERO, the research may have already been undertaken by another research group. Ideally, all journals should require that any work of this nature that is submitted should include a trial registration number and that editors should follow-up with the registry to ensure that similar work has not been previously planned and/or conducted. However, that is probably not realistic, would be difficult to enforce, and may stifle work that may be on the fringes of being similar, but in fact, may be quite different and/or unique. A second potential limitation that is not indigenous to this approach is the difficulty in determining when an updated systematic review is needed. While suggestions have been provided for such in this article, there is still a large degree of subjectivity involved. To a lesser extent, the same is true for a systematic review that has never been conducted on the topic of interest.

## Conclusions

The increasing number of systematic reviews, with or without meta-analysis, on the same topic now suggests that there is a need for a SRPSR as well as a decision regarding whether an original or updated systematic review, with or without meta-analysis, is appropriate. The suggested stepwise approach presented in this article provides a realistic way of addressing this and should be useful to anyone who is a producer or consumer of cancer-related systematic reviews. Ultimately, it is hoped that this work will contribute to improvements in evidence-based information aimed at the prevention and treatment of cancer.

## Additional files

Additional file 1: PRISMA protocol checklist for SRPRS (PRISMA-SRPSR-P). This file includes a modified PRISMA protocol checklist specific to systematic reviews of previous systematic reviews. (DOC 82 kb)

Additional file 2: PRISMA checklist for SRPRS. This file includes a modified PRISMA checklist specific to systematic reviews of previous systematic reviews (PRISMA-SRPSR). (DOC 60 kb)

Additional file 3: PRISMA protocol checklist. This file includes the PRISMA protocol checklist for systematic review protocols. (DOC 81 kb)

Additional file 4: PRISMA checklist for systematic reviews and meta-analyses. This file includes the PRISMA checklist for systematic reviews and meta-analyses, exclusive of network meta-analyses and IPD meta-analyses. (DOC 62 kb)

Additional file 5: PRISMA checklist for systematic reviews and network meta-analyses. This file includes the PRISMA checklist for systematic reviews and network meta-analysis. (DOCX 156 kb)

Additional file 6: PRISMA checklist for systematic reviews and IPD meta-analyses. This file includes the PRISMA checklist for systematic reviews and IPD meta-analyses. (DOCX 19 kb)

## Abbreviations

AMSTAR: A MeaSurement Tool to Assess systematic Reviews
GRADE: Grading of Recommendations, Assessment, Development and Evaluations
PRISMA: Preferred Reporting Items for Systematic Reviews and Meta-Analysis
ROBIS: Risk of Bias in Systematic Reviews
SRPSR: Systematic review of previous systematic reviews
