# Supplementary material for: Systematic reviews and cancer research: a suggested stepwise approach
Source: BMC Cancer. 2018 Mar 2;18:246. doi: 10.1186/s12885-018-4163-6 (PMC5834879; doi:10.1186/s12885-018-4163-6)
Supplement: Supplementary file 1 — PRISMA protocol checklist for SRPRS (PRISMA-SRPSR-P). This file includes a modified PRISMA protocol checklist specific to systematic reviews of previous systematic reviews. (DOC 82 kb) [file 12885_2018_4163_MOESM1_ESM.doc]

Additional file 1. Modified PRISMA protocol checklist for systematic reviews of previous systematic reviews (PRISMA-SRPSR-P).*

| Section and topic | Item No | Checklist item |
| --- | --- | --- |
| ADMINISTRATIVE INFORMATION | | |
| Title: |  |  |
| Identification | 1a | Identify the report as a protocol of a systematic review **of previous systematic reviews** |
| Update | 1b | If the protocol is for an update of a previous systematic review **of previous systematic reviews**, identify as such |
| Registration | 2 | If registered, provide the name of the registry (such as PROSPERO) and registration number |
| Authors: |  |  |
| Contact | 3a | Provide name, institutional affiliation, e-mail address of all protocol authors; provide physical mailing address of corresponding author |
| Contributions | 3b | Describe contributions of protocol authors and identify the guarantor of the **systematic review of previous systematic reviews** |
| Amendments | 4 | If the protocol represents an amendment of a previously completed or published protocol, identify as such and list changes; otherwise, state plan for documenting important protocol amendments |
| Support: |  |  |
| Sources | 5a | Indicate sources of financial or other support for the **systematic review of previous systematic reviews** |
| Sponsor | 5b | Provide name for the review funder and/or sponsor |
| Role of sponsor or funder | 5c | Describe roles of funder(s), sponsor(s), and/or institution(s), if any, in developing the protocol |
| INTRODUCTION | | |
| Rationale | 6 | Describe the rationale for the **systematic review of previous systematic reviews** in the context of what is already known |
| Objectives | 7 | Provide an explicit statement of the question(s) the **systematic review of previous systematic reviews** will address with reference, **where applicable** to participants, interventions, comparators, outcomes, **and settings/study designs (PICOS)** |
| METHODS | | |
| Eligibility criteria | 8 | Specify the study characteristics (such as PICO**S**, time frame) and report characteristics (such as years considered, language, publication status) to be used as criteria for eligibility for the **systematic review of previous systematic reviews** |
| Information sources | 9 | Describe all intended information sources (such as electronic databases, contact with study authors, trial registers or other grey literature sources) with planned dates of coverage |
| Search strategy | 10 | Present draft of search strategy to be used for at least one electronic database, including planned limits, such that it could be repeated |
| Study records: |  |  |
| Data management | 11a | Describe the mechanism(s) that will be used to manage records and data throughout the **systematic review of previous systematic reviews** |
| Selection process | 11b | State the process that will be used for selecting studies (such as two independent reviewers) through each phase of the **systematic review of previous systematic reviews** (that is, screening, eligibility and inclusion in meta-analysis) |
| Data collection process | 11c | Describe planned method of extracting data from reports (such as piloting forms, done independently, in duplicate), any processes for obtaining and confirming data from investigators |
| Data items | 12 | List and define all variables for which data will be sought (such as PICO**S** items, funding sources), any pre-planned data assumptions and simplifications |
| Outcomes and prioritization | 13 | List and define all outcomes for which data will be sought, including prioritization of main and additional outcomes, with rationale |
| **Quality and risk of bias of systematic reviews** | 14 | **Describe anticipated methods for assessing the quality, for example AMSTAR^a^, and/or risk of bias, for example, ROBIS^b^, of eligible systematic reviews** |
| Data synthesis | 15a | **Describe how summary findings for each outcome from each systematic review will be synthesised** |
|  | 15b | **Describe any additional analyses at the systematic review level not conducted in the original systematic review (influence analysis, cumulative meta-analysis, number-needed-to-treat, prediction intervals, Cohen’s U3 index, etc.)** |
|  | 15c | **Describe any proposed additional analyses, for example, one’s own meta-analysis based on studies nested within each systematic review, avoiding the inclusion of the same study from each systematic review more than once, calculation and pooling of effect sizes, assessment of heterogeneity, for example, Cochran’s Q statistic, assessment of inconsistency, for example I-squared, assessment of small-study effects, sensitivity analysis, including influence analysis, cumulative meta-analysis, number-needed-to-treat, prediction intervals, Cohen’s U3 index, meta-regression, etc.)** |
| Confidence in cumulative evidence | 16 | Describe how the strength of the body of evidence will be assessed (such as GRADE**^c^**) |

*** Boldfaced** items denote changes from the original PRSIMA-P checklist; ^a^, AMSTAR, A MeaSurement Tool to Assess systematic Reviews (see: Shea BJ, Hamel C, Wells GA, Bouter LM, Kristjansson E, Grimshaw J, Henry DA, Boers M: AMSTAR is a reliable and valid measurement tool to assess the methodological quality of systematic reviews. J Clin Epidemiol 2009, 62(10):1013-1020); ^b^, ROBIS, Risk of Bias in Systematic Reviews (see: Whiting P, Savovic J, Higgins JP, Caldwell DM, Reeves BC, Shea B, Davies P, Kleijnen J, Churchill R: ROBIS: A new tool to assess risk of bias in systematic reviews was developed. J Clin Epidemiol 2016, 69:225-234); ^c^, GRADE, Grading of Recommendations Assessment, Development and Evaluation (see: <http://www.gradeworkinggroup.org/>).

*Adapted From: Shamseer L, Moher D, Clarke M, Ghersi D, Liberati A, Petticrew M, Shekelle P, Stewart L, PRISMA-P Group. Preferred reporting items for systematic review and meta-analysis protocols (PRISMA-P) 2015: elaboration and explanation. BMJ. 2015 Jan 2;349(jan02 1):g7647.*
