# Supplementary material for: Systematic reviews and cancer research: a suggested stepwise approach
Source: BMC Cancer. 2018 Mar 2;18:246. doi: 10.1186/s12885-018-4163-6 (PMC5834879; doi:10.1186/s12885-018-4163-6)
Supplement: Supplementary file 2 — PRISMA checklist for SRPRS. This file includes a modified PRISMA checklist specific to systematic reviews of previous systematic reviews (PRISMA-SRPSR). (DOC 60 kb) [file 12885_2018_4163_MOESM2_ESM.doc]

Additional file 2. Modified PRISMA checklist for systematic reviews of previous systematic reviews (PRISMA-SRPSR).*

| **Section/topic** | **#** | **Checklist item** | **Reported on page #** |
| --- | --- | --- | --- |
| **TITLE** | | |  |
| Title | 1 | Identify the report as a systematic review of previous systematic reviews |  |
| **ABSTRACT** | | |  |
| Structured summary | 2 | Provide a structured summary including, as applicable: background; objectives; data sources; study eligibility criteria, participants, and interventions; study appraisal and synthesis methods; results; limitations; conclusions and implications of key findings; review registration number (**such as PROSPERO)** |  |
| **INTRODUCTION** | | |  |
| Rationale | 3 | Describe the rationale for the systematic review **of previous systematic reviews** in the context of what is already known |  |
| Objectives | 4 | Provide an explicit statement of questions being addressed with reference to participants, interventions, comparisons, outcomes, and study design (PICOS) |  |
| **METHODS** | | |  |
| Protocol and registration | 5 | Indicate if a review protocol exists, if and where it can be accessed (e.g., Web address), and, if available, provide registration information including registration number **(such as PROSPERO)** |  |
| Eligibility criteria | 6 | Specify study characteristics (e.g., PICOS, length of follow-up) and report characteristics (e.g., years considered, language, publication status) used as criteria for eligibility, giving rationale |  |
| Information sources | 7 | Describe all information sources (e.g., databases with dates of coverage, contact with study authors to identify additional studies) in the search and date last searched |  |
| Search | 8 | Present full electronic search strategy for at least one database, including any limits used, such that it could be repeated |  |
| Study selection | 9 | State the process for selecting studies (i.e., screening, eligibility, included in systematic review) |  |
| Data collection process | 10 | Describe method of data extraction from reports (e.g., piloted forms, independently, in duplicate) and any processes for obtaining and confirming data from investigators |  |
| Data items | 11 | List and define all variables for which data were sought (e.g., PICOS, funding sources) and any assumptions and simplifications made |  |
| **Quality and risk of bias of systematic reviews** | 12 | **Describe anticipated methods for assessing the quality, for example AMSTAR^a^, and/or risk of bias, for example, ROBIS^b^, of eligible systematic reviews** |  |
| Summary measures | 13 | State the principal summary measures (e.g., risk ratio, difference in means) |  |
| **Data** synthesis | 14a  14b  14c | **Describe how summary findings for each outcome from each systematic review were synthesised**  **Describe any additional analyses at the systematic review level not conducted in the original systematic review (influence analysis, cumulative meta-analysis, number-needed-to-treat, prediction intervals, Cohen’s U3 index, etc.**  **Describe any proposed additional analyses, for example, one’s own meta-analysis based on studies nested within each systematic review, avoiding the inclusion of the same study from each systematic review more than once, calculation and pooling of effect sizes, assessment of heterogeneity, for example, Cochran’s Q statistic, assessment of inconsistency, for example I-squared, assessment of small-study effects, sensitivity analysis, including influence analysis, cumulative meta-analysis, number-needed-to-treat, prediction intervals, Cohen’s U3 index, meta-regression, etc.** |  |

Page 1 of 2

| **Section/topic** | **#** | **Checklist item** | **Reported on page #** |
| --- | --- | --- | --- |
| **Confidence in cumulative evidence** | 15 | Describe how the strength of the body of evidence will be assessed (such as GRADE)^c^ |  |
| **RESULTS** | | |  |
| Study selection | 16 | Give numbers of **systematic reviews** screened, assessed for eligibility, and included in the review, with reasons for exclusions at each stage, ideally with a flow diagram |  |
| Study characteristics | 17 | For each **systematic review**, present characteristics for which data were extracted (e.g., study size, PICOS, follow-up period) and provide the citations |  |
| **Quality and risk of bias within systematic reviews** | 18 | Present data on **quality, for example AMSTAR^a^, and/or risk of bias, for example, ROBIS^b^, of eligible systematic reviews (see item 12)** |  |
| Synthesis of results | 19 | For all outcomes considered (benefits or harms), present, for **each systematic review**: (a) simple summary data and (b) effect estimates and confidence intervals, ideally with a forest plot Present results of each meta-analysis done, including confidence intervals and measures of consistency **(see item 14)** |  |
| **Confidence in cumulative evidence** | 20 | Report results on the strength of the body of evidence (such as GRADE)^c^ [see Item 15] |  |
| **DISCUSSION** | | |  |
| Summary of evidence | 21 | Summarize the main findings including the strength of evidence for each main outcome; consider their relevance to key groups (e.g., healthcare providers, users, and policy makers) |  |
| Limitations | 22 | Discuss limitations at study and outcome level (e.g., risk of bias), and at review-level (e.g., incomplete retrieval of identified research, reporting bias) |  |
| Conclusions | 23 | Provide a general interpretation of the results in the context of other evidence, and implications for future research |  |
| **FUNDING** | | |  |
| Funding | 24 | Describe sources of funding for the systematic review **of systematic reviews** and other support (e.g., supply of data); role of funders for the systematic review |  |

*** Boldfaced** items denote changes from the original PRSIMA-P checklist; a, AMSTAR, A MeaSurement Tool to Assess systematic Reviews (see: Shea BJ, Hamel C, Wells GA, Bouter LM, Kristjansson E, Grimshaw J, Henry DA, Boers M: AMSTAR is a reliable and valid measurement tool to assess the methodological quality of systematic reviews. J Clin Epidemiol 2009, 62(10):1013-1020); b, ROBIS, Risk of Bias in Systematic Reviews (see: Whiting P, Savovic J, Higgins JP, Caldwell DM, Reeves BC, Shea B, Davies P, Kleijnen J, Churchill R: ROBIS: A new tool to assess risk of bias in systematic reviews was developed. J Clin Epidemiol 2016, 69:225-234); c, GRADE, Grading of Recommendations Assessment, Development and Evaluation (see: <http://www.gradeworkinggroup.org/>).

*Adapted From:*  Moher D, Liberati A, Tetzlaff J, Altman DG, The PRISMA Group (2009). Preferred Reporting Items for Systematic Reviews and Meta-Analyses: The PRISMA Statement. PLoS Med 6(7): e1000097. doi:10.1371/journal.pmed1000097

Page 2 of 2
